# Newfound Coding Potential of Transcripts Unveils Missing Members of Human Protein Communities

**DOI:** 10.1016/j.gpb.2022.09.008

**Published:** 2022-09-30

**Authors:** Sébastien Leblanc, Marie A. Brunet, Jean-François Jacques, Amina M. Lekehal, Andréa Duclos, Alexia Tremblay, Alexis Bruggeman-Gascon, Sondos Samandi, Mylène Brunelle, Alan A. Cohen, Michelle S. Scott, Xavier Roucou

**Affiliations:** 1 Department of Biochemistry and Functional Genomics, Université de Sherbrooke, Sherbrooke, QC J1E 4K8, Canada; 2 PROTEO, Quebec Network for Research on Protein Function, Structure, and Engineering, Quebec City, QC G1V 0A6, Canada; 3 Department of Family Medicine, Université de Sherbrooke, Sherbrooke, QC J1H 5N4, Canada

**Keywords:** Alternative protein, Protein network, Protein–protein interaction, Pseudogene, Affinity purification mass spectrometry

## Abstract

Recent proteogenomic approaches have led to the discovery that regions of the transcriptome previously annotated as non-coding regions [*i.e.*, untranslated regions (UTRs), open reading frames overlapping annotated coding sequences in a different reading frame, and non-coding RNAs] frequently encode proteins, termed **alternative proteins** (**altProts**). This suggests that previously identified protein–protein interaction (PPI) networks are partially incomplete because altProts are not present in conventional protein databases. Here, we used the proteogenomic resource OpenProt and a combined spectrum- and peptide-centric analysis for the re-analysis of a high-throughput human network proteomics dataset, thereby revealing the presence of 261 altProts in the network. We found 19 genes encoding both an annotated (reference) and an alternative protein interacting with each other. Of the 117 altProts encoded by **pseudogenes**, 38 are direct interactors of reference proteins encoded by their respective parental genes. Finally, we experimentally validate several interactions involving altProts. These data improve the blueprints of the human PPI network and suggest functional roles for hundreds of altProts.

## Introduction

Cellular functions depend on myriads of protein–protein interaction (PPI) networks acting in consort and understanding cellular mechanisms on a large scale will require a relatively exhaustive catalog of PPIs. Hence, there have been major efforts to perform high-throughput experimental mapping of physical interactions between human proteins [1]. The methodologies involve binary interaction mapping using yeast 2-hybrid [2], biochemical fractionation of soluble complexes combined with mass spectrometry (MS) [3], and affinity purification mass spectrometry (AP-MS) [4], [5], [6].

In parallel to these experimental initiatives, computational tools were developed to help complete the human interactome [7]. Such tools are particularly useful for the identification of transient, cell type, or environmentally dependent interactions that escape current typical experimental protocols. Computational methods that can be used at large scales are created and/or validated using PPIs previously obtained experimentally [7], [8]. Thus, although computational tools complement experimental approaches, the experimental detection of PPIs is key to building a comprehensive catalog of interactomes.

The BioPlex network is the largest human proteome-scale interactome; initially, BioPlex 1.0 reporting 23,744 interactions among 7668 proteins was followed by BioPlex 2.0, which forms the basis of the current study, with 56,553 interactions reported involving 10,961 proteins. Recent pre-print BioPlex 3.0 reached 118,162 interactions among 14,586 proteins in HEK293T cells [4], [5], [9]. The enrichment of interactors of roughly half of currently annotated (or reference) human proteins allowed the authors to functionally contextualize poorly characterized proteins, identify communities of tight interconnectivity, and find associations between disease phenotypes and these protein groups. Here, a community represents a group of nodes in the network that are more closely associated with themselves than with any other nodes in the network as identified with an unsupervised clustering algorithm. In addition, pre-print BioPlex now provides a first draft of the interactome in HCT116 cells [9].

**Figure 1:**
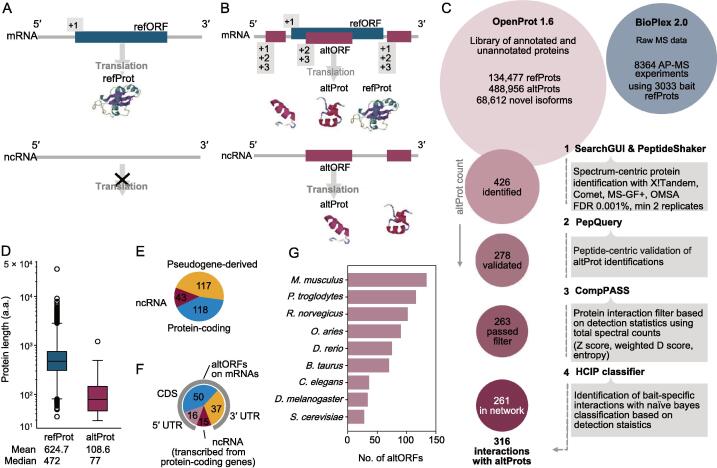
**Analysis overview and identification of altProts in the human interactome** **A.** and **B.** The classical model of RNA transcript CDS annotation includes only one refORF on mRNAs encoding a refProt and no functional ORF within ncRNAs (A), whereas the alternative translation model considers multiple proteins encoded in different reading frames in the same transcript including refProts and altProts (B). **C.** Our re-analysis pipeline of high-throughput AP-MS experiments from BioPlex 2.0 employs stringent criteria to ensure confident identification of both protein detection and interaction detection. Of the 426 altProts initially identified in the dataset, 261 joined the network of protein interactions after filtration. **D.** altProts are in general shorter than refProts. Boxes represent the inter quartile range marked at the median and the whiskers are set at 1.5 times inter quartile range over and under the 25th and 75th percentiles. **E.** Identified altProts (278) were encoded by transcripts of a variety of biotypes. 118 of identified altProts are encoded by transcripts of protein-coding biotype, 117 by transcripts of pseudogenes, and 43 exclusively by transcripts of non-coding biotype. **F.** altORFs found to be encoded by transcripts from genes of protein-coding biotype are most often overlapping the canonical CDS or localized downstream in the 3′ UTR. A significant fraction of altORFs also localize in ncRNAs of protein-coding genes. **G.** Orthology data across 10 species from OpenProt 1.6 for detected altProts. altProt, alternative protein; ORF, open reading frame; refProt, reference protein; altORF, alternative ORF; refORF, reference ORF; AP-MS, affinity purification mass spectrometry; ncRNA, non-coding RNA; CDS, coding sequence; UTR, untranslated region; FDR, false discovery rate; HCIP, high-confidence interacting protein.

The experimental strategy behind BioPlex is based on the expression of each protein-coding open reading frame (ORF) present in the human ORFeome with an epitope tag, the affinity purification of the corresponding protein, and the confident identification of its specific protein interactors by MS. The identification of peptides and proteins in each protein complex is performed using the UniProt database. Hence, only proteins and alternative splicing-derived protein isoforms annotated in the UniProt database can be detected. Using this common approach, the human interactome is necessarily made up of proteins already annotated in the UniProt database, precluding the detection of novel unannotated proteins. Yet, beyond isoform-derived proteomic diversity, multiple recent discoveries point to a general phenomenon of translation events of non-canonical ORFs in both eukaryotes and prokaryotes, including small ORFs and alternative ORFs (altORFs) [10], [11], [12]. Typically, small ORFs are between 10 and 100 codons, whereas altORFs can be larger than 100 codons. Here, we use the term altORFs for non-canonical ORFs independently of their size. On average, altORFs are ten times shorter than conventional annotated ORFs, but several thousands are longer than 100 codons [13]. altORFs encode alternative proteins (altProts) and are found both upstream (*i.e.*, 5′ UTR) and downstream (*i.e.*, 3′ UTR) of the reference coding sequence (CDS) as well as overlapping the reference CDS in a shifted reading frame within mRNAs (Figure 1A and B). Additionally, RNAs transcribed from long non-coding RNA genes and pseudogenes are systematically annotated as non-coding RNAs (ncRNAs); yet, they may also harbor altORFs and encode altProts [13]. Consequently, the fraction of multi-coding or polycistronic human genes and of protein-coding “pseudogenes” may have been largely underestimated. altORFs translation events are experimentally detected by ribosome profiling [11], a method that detects initiating and/or elongating ribosomes at the transcriptome wide level [14]. Alternatively, large-scale MS detection of altProts requires first the annotation of altORFs and then *in silico* translation of these altORFs to generate customized protein databases containing the sequences of the corresponding proteins [15]. This integrative approach, termed proteogenomics, has emerged as a new research field essential to better capture the coding potential and the diversity of the proteome [16], [17].

The translation of altORFs genuinely expands the proteome, and proteogenomics approaches using customized protein databases allows for routine MS-based detection of altProts [18], [19]. In order to uncover altProts otherwise undetectable using the UniProt database we re-analyzed the raw MS data from the BioPlex 2.0 interactome with our OpenProt proteogenomics database.

OpenProt contains the sequences of proteins predicted to be encoded by all ORFs larger than 30 codons in the human transcriptome. This large ORFeome includes ORFs encoding proteins annotated by NCBI RefSeq, Ensembl, and UniProt, termed here reference proteins (refProts). It also includes still unannotated ORFs that encode novel isoforms sharing a high degree of similarity with refProts from the same gene. Finally, the third category of ORFs, termed altORFs, potentially encode altProts and share no significant sequence similarity with a refProt from the same gene (Table 1). OpenProt is not limited by the three main assumptions that shape current annotations: (1) a single functional ORF in each mRNA, typically the longest ORF; (2) RNAs with ORFs shorter than 100 codons are typically annotated as ncRNAs; and (3) RNAs transcribed from genes annotated as pseudogenes are automatically annotated as ncRNAs. Thus, in addition to proteins present in NCBI RefSeq, Ensembl, and UniProt, OpenProt also contains the sequence for novel proteins, including novel isoforms and altProts [20], [21]. Using OpenProt, we were able to detect and map altProts within complexes of known proteins which increased protein diversity by including a higher number of small proteins. In addition, the data confirmed the significant contribution of pseudogenes to protein networks with 117 out of 261 altProts encoded by genes annotated as pseudogenes. We also detected many interacting proteins encoded either by the same gene or by a pseudogene and its corresponding parental gene. In sum, this work improves our knowledge of both the coding potential of the human transcriptome and the composition of protein communities by bringing diversity (*i.e.*, small proteins) and inclusivity (*i.e.*, proteins encoded in RNAs incorrectly annotated as ncRNAs) into the largest human PPI network to date.

**Table 1:** **Terminology definitions**

**Terminology**	**Definition**
ORF	Sequence of nucleotides bounded by start and stop codons potentially translated into protein by ribosomes
refORF	Annotated ORF producing a known protein
altORF	Unannotated ORF producing an unknown/unannotated protein; altORFs can be found on mRNAs overlapping refORFs or in untranslated regions, or on ncRNAs
refProt	Annotated protein product resulting from the translation of a refORF
altProt	Unannotated protein product resulting from the translation of an altORF with no significant homology with any refProt from the same gene
Novel isoform	Unannotated protein product resulting from the translation of an altORF with high homology to a refProt from the same gene

*Note*: ORF, open reading frame; refORF, reference ORF; altORF, alternative ORF; refProt, reference protein; altProt, alternative protein; ncRNA, non-coding RNA.

## Results

### Re-analysis of BioPlex 2.0 MS data and identification of preyed altProts

We employed the OpenProt proteogenomic library in the re-analysis of high-throughput AP-MS experiments from the BioPlex 2.0 network. Given the size of the OpenProt database (Figure 1C), the false discovery rate (FDR) for protein identification was adjusted from 1% down to 0.001% to mitigate against spurious identifications [20]. Such stringent FDR settings inevitably lead to fewer prey proteins identified; thus, our highly conservative methodology is likely to leave behind many false negatives. The BioPlex 2.0 network is built in a gene-centric manner to simplify the analysis by making abstraction of protein isoforms. In the current analysis, all refProts and their isoforms are also grouped under their respective gene, but results concerning altProts are necessarily given at the protein level.

**Supplementary Figure S1:**
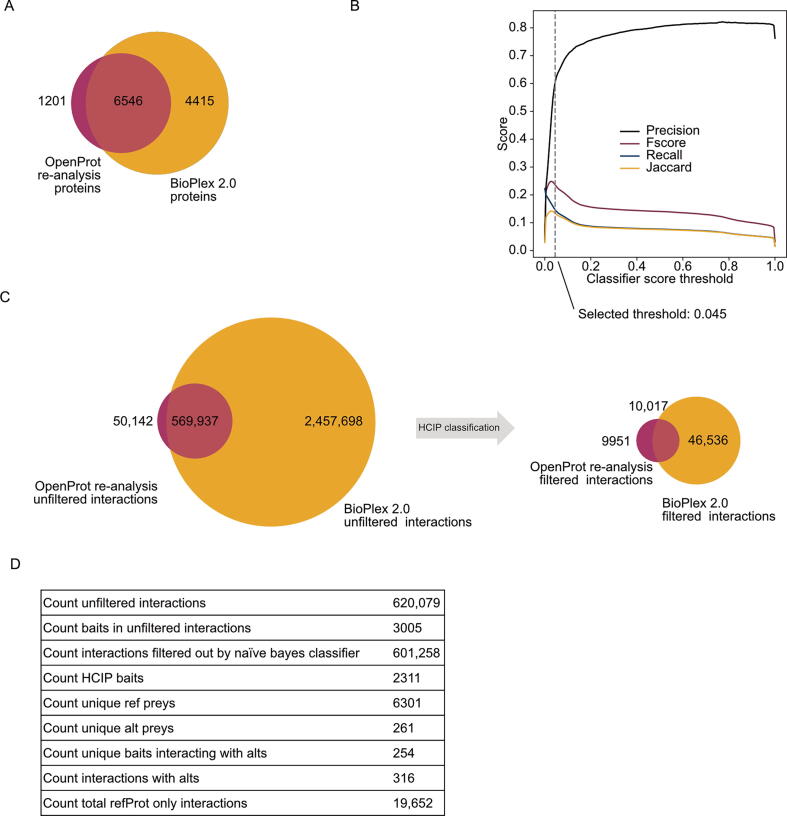
**Network assembly details A.** Overlap of total proteins (nodes) in BioPlex 2.0 and OpenProt derived networks. **B.** Classifier performance across thresholds. Scores were computed using the BioPlex 2.0 network as ground truth. **C.** The overlap of unfiltered interactions between BioPlex 2.0 and the result of OpenProt 1.6 derived re-analysis was considerable (92% of re-analysis candidate PPIs) (i). Upon filtration the overlap is still significant despite the marked smaller size of the OpenProt derived network (59% of re-analysis PPIs). **D.** Detailed counts of protein and interaction identifications.

In total, 426 unannotated proteins from 414 genes and 8972 refProts were identified in the re-analysis of raw MS data from the pull-down of 3033 refProts (baits), using a combination of multiple identification algorithms (Figure 1C). Because these identifications resulted from the re-analysis of raw MS data from BioPlex 2.0 with the OpenProt MS pipeline, we sought to determine the overlap between total sets of genes identified. refProts from 6546 genes (or 84% of total re-analysis results) were found in both BioPlex 2.0 and in the present work (Figure S1A), indicating that the re-analysis could reliably reproduce BioPlex results.

**Supplementary Figure S2:**
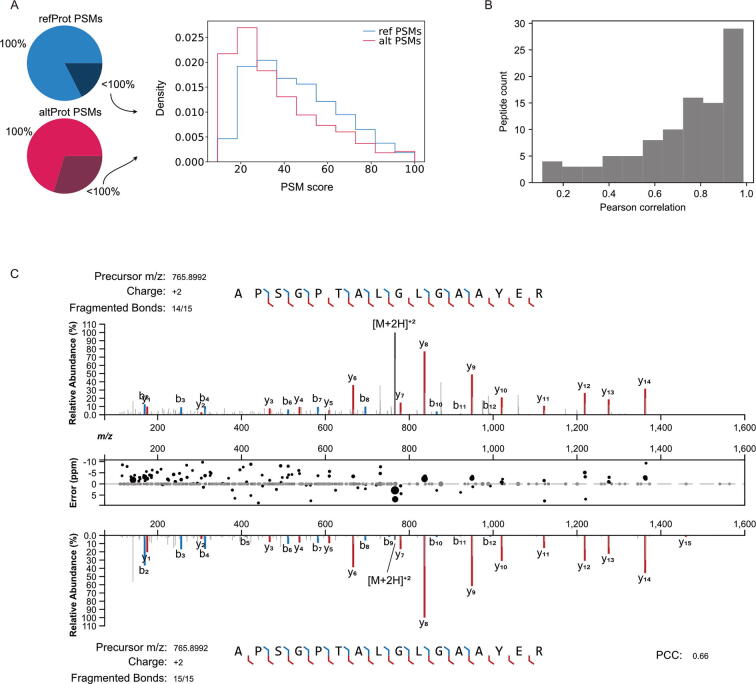
**PSM score distributions and validation of novel peptide fragmentation A.** Distribution of PSM scores involving peptides of refProts (blue) and altProts (red). The histogram shows the distribution of scores for those below 100%. PSM scoring is a combination of scores from all search algorithms via SearchGUI and is outputted as a confidence level. **B.** Distribution of Pearson correlation scores indicating the degree to which spectra obtained from synthetic peptides were found contained in spectra observed in the BioPlex data. 100 peptides from altProts were synthesized and analyzed under an MS/MS regime similar to BioPlex to validate the fragmentation pattern of observed spectra (see Methods). 98 synthetic peptides produced spectra correlating with the spectra of the corresponding peptides observed in the BioPlex data. 2 synthetic peptides were not detected in the analysis and were excluded from the comparisons. **C.** Top: spectrum observed in the BioPlex data (spectrum title: j6218.11202.11202.2) assigned to peptide APSGPTALGLGAAYER uniquely mapping to the altProt IP_250569 encoded by the long non-coding RNA gene AC004706.3. Bottom: spectrum obtained from MS/MS analysis of the synthetic peptide APSGPTALGLGAAYER. The two spectra have a PCC of 0.66 which places the comparison near the median for all peptides. The majority of comparisons between observed and synthetic spectra is therefore of equal or better quality to that of APSGPTALGLGAAYER. alt, alternative; ref, reference; PSM, peptide spectrum match; PCC, Pearson correlation coefficient.

Although peptide spectrum match (PSM) scores of altProt peptides tended to be slightly lower than those of refProt on average, the overall distributions were similar (Figure S2A). For this reason, our stringent approach in the identification of altProts included the use of PepQuery to validate protein detection using a peptide-centric approach [22]. This tool includes a step which verified that altProt-derived peptides were supported by experimental spectra that could not be better explained by peptides from refProts with any post-translational modification. In addition, peptides were screened for isobaric substitutions in order to reject dubious peptides that could match refProts [23]. A total of 278 altProt identifications were validated with PepQuery including 136 altProts encoded by pseudogenes (Figure 1C, Figure S1; Table S1).

The observed fragmentation pattern of peptides was validated through MS/MS analysis of 100 synthetic peptides from 72 altProts encoded by transcripts of various biotypes. The spectral correlation coefficient was computed between spectra observed in BioPlex and those of synthetic peptides and 74 of these showed coefficients higher than 0.6 (Figure S2B; Table S2). An example comparison of spectra with correlation coefficient of 0.66 is shown in Figure S2C (median correlation coefficient across comparisons of 0.78). These results confirmed that spectra assigned to altProt peptides were representative of the fragmentation pattern obtained from corresponding synthetic peptides.

MS-based identification of short proteins with a minimum of 2 unique suitable tryptic peptides remains an important challenge and most of short proteins are typically detected with a single unique peptide [24], [25]. Of the 278 altProts validated by PepQuery (Table S1), 68 complied with the Human Proteome Project PE1 level for proteins with strong protein-level evidence, Guidelines v3.0 [26]. Apart from their detection in the BioPlex dataset, 156 were also detected in other MS datasets and 18 showed evidence of translation via ribosome profiling (Table S1). In addition, 27 of detected altProts were reported by the SmProt resource and 5 were present in the sORFs library (Table S1) [12], [27].

As expected, detected altProts were much shorter than refProts with a median size of 77 aa *versus* 472 aa (Figure 1D; Table S1). It is well known that small proteins suffer from less sequence coverage in MS/MS analysis [28], [29] and this was also observed in the current study. The average detected sequence coverage was 41% for refProts and 23% for altProts. This is only considering peptides that are unique to altProts. If peptides matching both a refProt and an altProt were detected, they were not considered as evidence for the expression of the altProt and so were excluded from the coverage calculation. Pseudogene products particularly are usually identified with a small number of peptides (sometimes only one), because other peptides are shared with the protein from the parental gene.

altORFs encoding the 278 detected and PepQuery-validated altProts were distributed among 971 transcripts (Table S1), and in addition to the 117 pseudogene-derived altProts, 43 were exclusively encoded by genes of non-coding biotypes (Figure 1E). A third were found in transcripts already encoding a refProt (Figure 1E), indicating that the corresponding genes are in fact either bicistronic (two non-overlapping ORFs) or dual-coding (two overlapping ORFs) (Table S1). Of the altProts encoded by transcripts from genes of protein-coding biotype, most were encoded by a frame-shifted altORF overlapping the annotated CDS or downstream of the annotated CDS in the 3′ UTR (Figure 1F). The remaining altORFs were encoded by 5′ UTRs or by transcripts annotated as non-coding but transcribed from those genes of protein-coding biotype. From the localization of altORFs relative to the canonical CDS in the mRNA from protein-coding genes, we conclude that 70 of those genes are in fact bicistronic and 56 are dual-coding (Table S1). In addition, transcripts from three pseudogenes have been found to encode two altProts suggesting that they are in fact bicistronic (Table S1).

We collected protein orthology relationships from 10 species computed by OpenProt (Figure 1G). Although 100 altProts were specific to humans, a large number had orthologs in the mouse and chimpanzee, and 28 were even conserved through evolution because 116 yeast altProts displayed at least one functional domain signature (InterProScan, version 5.14–53.0, [30]), further supporting their functionality (Table S1).

### Assembling PPIs into a network

After identification of prey proteins, CompPASS was used to compute semi-quantitative statistics based on PSM across technical replicates [31]. These metrics allow filtration of background and spurious interactions from the raw identifications of prey proteins to obtain high-confidence interacting proteins (HCIPs). To mitigate against the otherwise noisy nature of fast-paced high-throughput approaches and to filter prey identifications down to the most confident interactions, we applied a Naïve Bayes classifier similar to CompPASS Plus [4]. The classifier used representations of bait–prey pairs computed from detection statistics and assembled into a vector of 9 features as described by Huttlin and his colleagues [4]. High confidence interactions reported by BioPlex 2.0 served as target labels. HCIP classification resulted in the retention of 3.2% of the starting set of bait–prey pairs identified (Figure S1C). Notably, 694 baits from the original dataset were excluded after filtration because no confident interaction could be distinguished from background.

**Figure 2:**
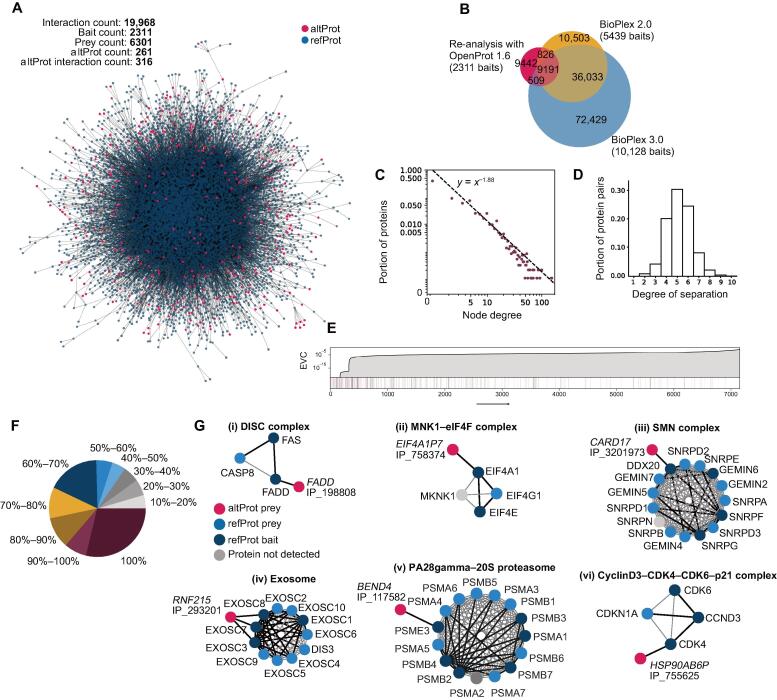
**Interaction mapping and network features ofPPIs** **A.** The largest component of the network assembled from the OpenProt-based re-analysis of high-throughput AP-MS data from BioPlex 2.0. **B.** A venn diagram of bait–prey interactions identified with the OpenProt-derived re-analysis, BioPlex 2.0, and BioPlex 3.0 shows a significant overlap despite the smaller overall size of the re-analysis results (due to stringent filtration). It should also be noted that altProts were not present in the BioPlex 2.0 analytical pipeline which accounts for part of the gap in overlap. **C.** The degree distribution (distribution of node connectivity) follows a power law as demonstrated by a discrete maximum likelihood estimator fit. Most of the proteins have a small number of connections, whereas a few are highly connected (often called hubs). **D.** The distribution of degrees of separation between all protein pairs (*i.e.*, the length of the shortest path between all pairs of proteins) indicates that the network fits small-world characteristics. **E.** altProts were found diffusely throughout the network and across the spectrum of EVC (dark lines). EVC is a relative score that indicates the degree of influence of nodes on the network; here, altProts display involvement in both influential and peripheral regions. **F.** Known protein complexes from the CORUM 3.0 resource (Giurgiu et al. [75]) were mapped onto the network. Subunit recovery rate confirms the overall validity of the interactions confidently identified by the pipeline. All CORUM core complexes for which at least two subunits appear as baits in the network were considered. **G.** Selected CORUM complexes are shown with the addition of altProts found in the interaction network of baited subunits. Black edges indicate detection in the re-analysis, and gray edges indicate those only reported by CORUM. PPI, protein–protein interaction; EVC, eigenvector centrality.

Following protein identifications and background filtration, the network was assembled by integrating all bait–prey interactions into one network (Figure 2A). All refProts and their isoforms were grouped under their respective gene, similar to the BioPlex analysis, but separate nodes are shown for altProts. In total, the re-analysis with OpenProt found 6301 prey proteins from the purification of 2311 bait proteins altogether engaged in 19,968 interactions, 51% of which were also reported by BioPlex 2.0 (Figure 2B). The average number of interactions per bait was 9.7. Among prey proteins, 261 altProts were found engaged in 316 interactions with 292 bait proteins.

Compared with BioPlex 2.0, a smaller total number of protein identification was expected because the OpenProt MS analysis pipeline is more stringent with a tolerance of 20 ppm on peak positions rather than 50 ppm and a 0.001% protein FDR as opposed to 1%. Indeed, we identified 19,968 interactions in our re-analysis, compared with 56,553 interactions reported by BioPlex 2.0 (Figure 2B). Among the 19,968 interactions, 10,017 (51%) were also reported by BioPlex 2.0, and 9700 (49%) were reported in the recently released BioPlex 3.0 (Figure 2B). Interestingly, 11,329 interactions (20%) from BioPlex 2.0 were not confirmed in BioPlex 3.0 using a larger number of protein baits, although the same experimental and computational methodologies were used (Figure 2B). This observation illustrates the challenge in the identification of PPIs with large-scale data given the relatively low signal to noise ratio in AP-MS data.

### Network structural features and altProt integration

Network theoretic analysis confirmed that the OpenProt-derived network displayed the expected characteristics of natural networks. Variability in the number of interacting partners of a given protein in a network (node degree) is typically very wide and the degree distribution that characterizes this variation follows a power law [32]. Similar to other protein networks, the degree distribution of the OpenProt-derived network also fitted a power law, an indication that most of the proteins have few connections and a minor fraction is highly connected (also called hubs) (Figure 2C). The degree of connectivity of altProts varied between 1 and 5 whereas that of refProt was between 1 and 179. On the one hand, because long and multidomain proteins are over-represented among hub proteins [33], this difference may be explained by the fact that altProts in the network were on average 6 times shorter than refProts (Figure 1D). On the other hand, none of the altProts were used as baits which also explains their lower observed connectivity because average degree was 1.2 for preys but 5.3 for baits.

The mean degrees of separation between any two proteins in the OpenProt-derived network was 5 (Figure 2D), in agreement with the small-world effect that characterizes biological networks [34].

Centrality analysis allows sorting proteins according to their relative influence on network behavior in which the most central proteins tend to be involved in the most essential cellular processes [35]. Here, the eigenvector centrality (EVC) measure indicates that altProts are found both at the network periphery connected to refProts of lesser influence as well as connected to central refProts of high influence (Figure 2E). Because no altProts were used as baits, they are likely artificially pushed toward the edges of the network. Known complexes from the CORUM database were mapped onto the network to assess the portion of complex subunits identified in the re-analysis (Table S3). In most cases a majority were recovered (75% of complexes showed ≥ 50% recovery) (Figure 2F). We observed 33 altProts in the neighborhood of CORUM complex subunits that served as bait, *i.e.*, directly interacting with the CORUM complex. Here multiple interesting patterns of altProt interactions were already noticeable: (1) altProts detected in the interactome of their respective refProts (Figure 2G, i), (2) altProts originating from pseudogenes and detected in the interactome of refProts encoded by the parental gene (Figure 2G, ii), and (3) altProts from protein-coding genes or pseudogenes detected in network regions outside the immediate neighborhood of the related protein/gene (Figure 2G, iii–vi).

**Figure 3:**
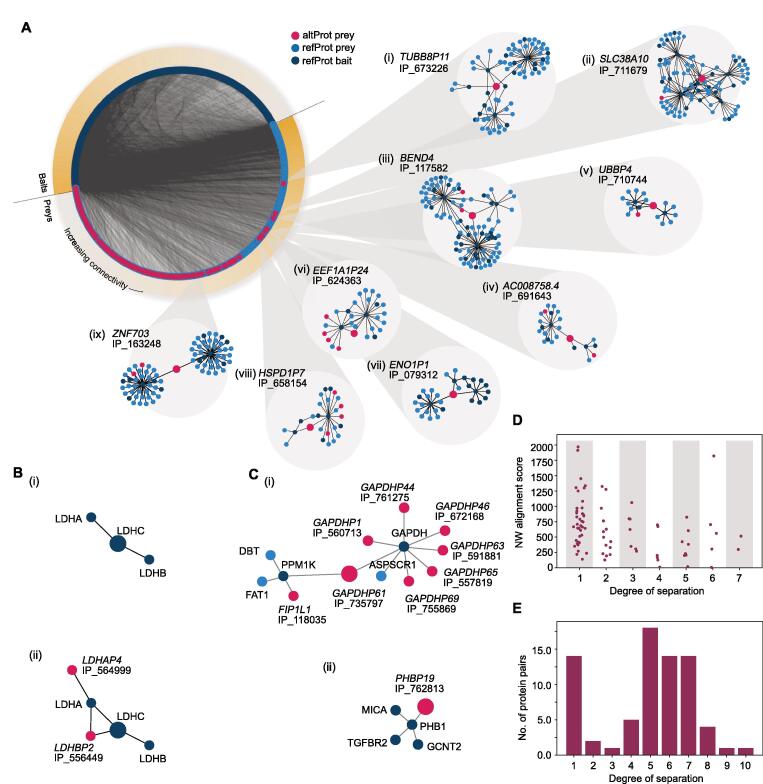
**Specific features ofPPIs involving preyed altProts** **A.** Degree-sorted circular layout of the OpenProt-derived full network separated by bait and preys. Direct neighbors and neighbors of neighbors (here called second neighborhood) were extracted for each altProt. Second neighborhoods of altProts display a variety of topologies with some acting as bridges (iv, v, vii, and ix) and others embedded in interconnected regions (i–iii and vi). Larger nodes represent the proteins for which the second neighborhood was extracted. **B.** Second neighborhood of the refProt LDHC extracted from the network assembled without altProts (i) and with altProts (ii). Inclusion of altProts in the network revealed that LDHC second degree network contains two proteins encoded by pseudogenes of the LDH family. Larger nodes represent the proteins for which the second neighborhood was extracted. **C.** Detailed second neighborhood of two pseudogene-encoded altProts. (i) GAPDH refProt shows 9 altProt interactors encoded by pseudogenes of GAPDH. (ii) AltProt encoded by *PHBP19* seen in the neighborhood of the PHB refProt. Larger nodes represent the proteins for which the second neighborhood was extracted. **D.** altProt found in the direct interactome of corresponding refProt from parental genes display a wide array of sequence similarity to the refProt. Pairs of altProt–refProt from pairs of pseudogene–parental gene are slightly closer in the network if their NW protein sequence global alignment score is higher. **E.** The distribution of degrees of separation between altProt–refProt pairs of the same gene is bimodal with a sub-population (75%) following a distribution similar to the full network (see Figure 2D), and the other placing altProts in the direct neighborhood of refProts from the same gene. NW, Needleman–Wunch.

The OpenProt-derived PPI network displayed with a degree sorted circle layout showed that preyed altProts generally had a lower degree of connectivity compared with refProts (Figure 3A). This might be expected in part because no altProts were used as baits in the network, but also based on the limited range of binding capacity due to their smaller size. In order to investigate the local neighborhood of altProts, subnetworks were extracted by taking nodes within shortest path length of 2 and all edges between these for each altProt (here called second neighborhood). The most connected altProt is a product of a tubulin pseudogene (Figure 3A, i). Other notable altProts with high degree include OpenProt accessions IP_711679, encoded in a transcript of the *SLC38A10* gene currently annotated as a ncRNA (Figure 3A, ii), and IP_117582, a novel protein encoded by an altORF overlapping the reference CDS in the *BEND4* gene (Figure 3A, iii). Although these two altProts would not qualify as hub proteins per say, they seem to participate in the bridging of hubs from otherwise relatively isolated regions. Several other examples of altProts encoded by a lncRNA gene (Figure 3A, iv), in pseudogenes (Figure 3A, v–viii), and in protein-coding genes (Figure 3A, iv and ix) integrate the network with a variety of topologies. One of these subnetworks features IP_710744, a recently discovered altProt and polyubiquitin precursor with three ubiquitin variants, was encoded in the *UBBP4* pseudogene [36]. The ubiquitin variant Ubbp4^A2^ differs from canonical ubiquitin by one amino acid (T55S) and can be attached to target proteins [36]. Before network assembly this variant was identified reproducibly (across technical replicates) in the purification of 11 baits. Following HCIP identifications, only three interactions remained (Figure 3A, v), likely because widespread identifications lead the Naïve Bayes classifier to assume non-specificity for those showing lower abundance. The three interactors include two ubiquitin ligases, *UBE2E2* (Q96LR5) and *UBE2E3* (Q969T4), and *USP48* (Q86UV5), a peptidase involved in the processing of ubiquitin precursors.

After observing second neighborhoods of altProts we sought to evaluate the effect of altProt inclusion into local neighborhoods of refProts. To do so, we computed the EVC of each refProt within their own second neighborhood extracted from the assembled network with and without altProts. This analysis highlighted *LDHC* which undergoes a marked increase in EVC in its second neighborhood (1.0 *versus* 0.5) when the altProts IP_556449 and IP_564999 (both pseudogenes of the LDH family) are included (Figure 3B, i and ii). This shows that node influence in this region of the network is impacted by the presence of altORF products.

In total, 39 pseudogene-encoded altProts were uncovered in the direct interactome of refProts from their respective parental genes (Table S4, shortest path length of 1), of which two more examples are illustrated with more details in Figure 3C.

**Supplementary Figure S3:**
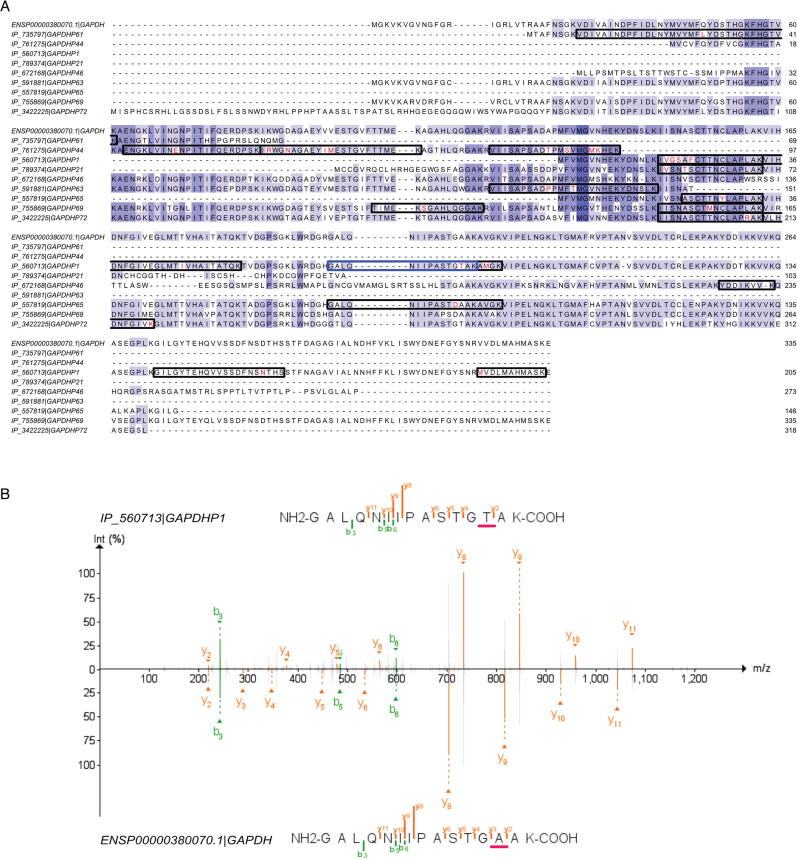
**GAPDH pseudogenes identified with unique peptides A.** Alignement of GAPDH refProt sequence with the altProt sequences derived from pseudogenes of GAPDH. Blue color represents the level of identity between sequences per amino acid. Boxed subsequences show the peptides detected in the BioPlex 2.0 raw data. Amino acids colored in red highlight the differences with the refProt sequence. A spectrum associated to the peptide boxed in blue is shown in panel B. **B.** Mass spectrum from the BioPlex 2.0 (file name: j6172.raw, spectrum title: j6172.8294.8294.2) shows the presence of two similar peptides. All four spectrum matching algorithms assigned the spectrum to the pseudogene derived altProt but shifted peaks of y fragment ions suggested the presence of a similar peptide from the refProt with a single amino acid difference (T>A). Peak annotations in green correspond to the y ions of the refProt peptide.

*GAPDH* is known to have a large number of pseudogenes [37]. Yet protein products originating from seven *GAPDH* pseudogenes were confidently identified in the purification of the canonical GAPDH protein (Figure 3C, i). Because the glycolytic active form of this enzyme is a tetramer, we conjecture that GAPDH tetramers may assemble from a heterogenous mixture of protein products from the parental gene and many of its pseudogenes. GAPDH is a multifunctional protein [38]; although different posttranslational modifications may explain in part how this protein switches function [39], it is possible that heterologous and homologous complexes contribute to GAPDH functional diversity. This is supported by the fact that 4 of the smallest protein products from *GAPDH* pseudogenes only contain the GAPDH NAD binding domain (IPR020828; IP_735797, IP_761275, IP_735800, IP_591881); the protein encoded by *GAPDHP1* only contains the GAPDH catalytic domain (IPR020829; IP_560713); whereas the largest proteins from *GAPDH* pseudogenes contain both domains (IP_557819, IP_672168, IP_3422225, IP_755869) (Table S1). The *PHB1* subnetwork highlights an interaction between *PHB1* and *PHBP19*, one of the 21 *PHB* pseudogenes (Figure 3B, ii). *PHB1* and *PHB2* are paralogs and the proteins they encode, PHB1 and PHB2, heterodimerize; similar to GAPDH, the PHB1/PHB2 complex is multifunctional [40], and the dimerization of PHB1 or PHB2 with *PHBP19*-derived IP_762813, which also contains a prohibitin domain (IPR000163), may regulate the various activities of the complex. Each GAPDH pseudogene identification is supported by a unique peptide (Figure S3A). Whereas most peptides differ by one or two amino acids with the canonical sequence, the spectrum in Figure S3B clearly shows the presence of co-eluding peptides of GAPDH and GAPDHP1 and was likely assigned to the refProt in the BioPlex analysis, but received a better score with the pseudogene in the OpenProt-derived analysis.

We reasoned that pseudogene-derived altProts directly interacting with their parental gene-derived refProts (parental protein) may result from the generally high degree of sequence similarity, particularly for refProts known to multimerize. However, although a slight reduction of alignment scores was observed with an increase in degrees of separation, the 39 altProts directly interacting with parental protein display a large variety of sequence alignment scores (Figure 3D). This suggests that direct interactions between pseudogene-derived altProts and their respective parental refProts involve other mechanisms in addition to sequence identity. Because 37 of the 39 altProts share between 1 and 6 InterPro entries with their respective parental proteins (Table S1), protein domains may be an important mechanism driving these interactions.

The mean degrees of separation between a refProt and an altProt encoded in the same gene reveals two types of relationships (Figure 3E). 19% (14) of altProt–refProt pairs have a degree of separation of 1, that is to say these altProts were found in the direct interactome of the corresponding refProt from the same gene (Table S4). Hence, these protein pairs encoded by the same genes are clearly involved in the same function through direct or indirect physical contacts. Interestingly, 12 of these 14 altProts are encoded by dual-coding genes, *i.e.*, with altORFs overlapping annotated CDSs. The remaining altProt–refProt pairs follow a distribution of degrees of separation similar to the whole network (compare Figures 3E and 2D). This suggests that they are not more closely related than any random pair of proteins in the network despite shared transcriptional regulation.

### Cluster detection reveals altProts as new participants in known protein communities

**Figure 4:**
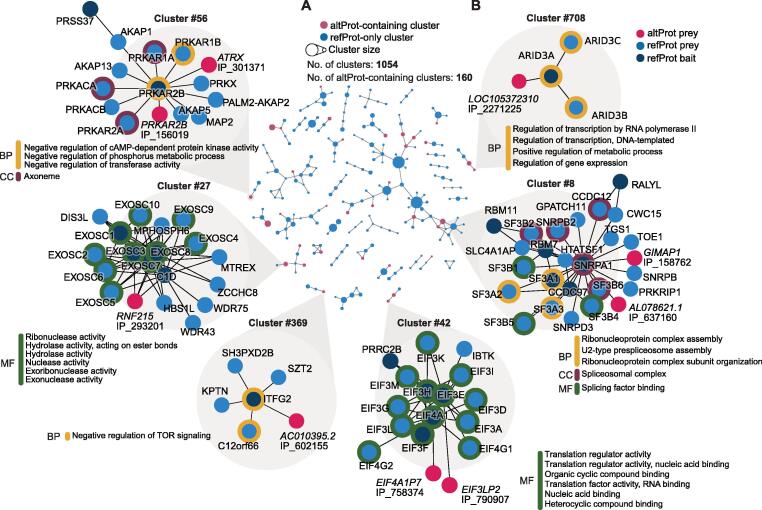
**Protein communities obtained via unsupervised community detection reveal new members** **A.** Protein communities identified via the Markov clustering algorithm (Enright et al. [41]). A total of 1054 clusters and 266 connections between them were identified; however, here are shown only components of three clusters or more for brevity. Nodes represent protein clusters sized relative to the number of proteins. Connections between clusters were determined by calculating enrichment of links between proteins in pairs of clusters using a hypergeometric test with maximal alpha value of 0.05 and correction for multiple testing was applied with 1% FDR. **B.** Focus on selected clusters showing significant enrichment of GO terms. Enrichment was computed against background of whole genome with alpha value set to < 0.05 and Benjamini–Hochberg corrected FDR of 1%. GO, Gene Ontology; BP, biological process; MF, molecular function; CC; cellular compartment.

Biological networks are organized in a hierarchy of interconnected subnetworks called clusters or communities. To identify these communities, unsupervised Markov clustering [41] was used similarly to methodology applied to BioPlex 2.0 [5]. Partitioning of the network resulted in 1054 protein clusters, 160 of which contained at least one altProt (Figure 4A). The size of altProts in these communities varied between 29 to 269 aa indicating that protein length may not be a limiting factor in their involvement in functional groups. Links between clusters were drawn in which the number of connections between members of cluster pairs was higher than expected (detailed in Materials and methods).

In order to assign biological function to these clusters, and therefore generate testable hypotheses about the function of altProts detected among them, enrichment of Gene Ontology (GO) terms was computed for each community against the background of all human genes. Several communities of different sizes showing significant GO term enrichment are detailed in Figure 4B.

About 50% of identified clusters showed GO term enrichment. The same analysis with the original BioPlex network showed 57% of clusters with GO term enrichment; possibly because a higher number of protein identifications yielded a larger network and therefore a higher probability of significant enrichment.

The altProt IP_293201 from the gene *RNF215* was identified as a novel interactor of three subunits of the RNA exosome multisubunit complex (cluster #27), suggesting a possible role in RNA homeostasis. Clusters #42 and #369 included protein communities with essential activities: the large eukaryotic initiation factor EIF3 and the recently discovered KICSTOR complex, a lysosome-associated negative regulator of mTORC1 signaling [42]. At least one pseudogene encoded altProt was detected in each of these clusters. Intriguingly, altProts IP_790907 (cluster #42) and IP_602155 (cluster #369) interact with the parental proteins EIF3E and ITFG2, respectively. These altProts may either compete with the parental proteins to change the activity of the complexes, or function as additional subunits because each contains a relevant functional domain (initiation factor domain IPR019382 and ITFG2 domain PF15907, respectively). Several subunits of the spliceosome are present in cluster #8, a protein community that includes IP_637160, a novel interactor of SNRPA1, which contains a U2A'/phosphoprotein 32 family A domain (IPR003603) where U2A' is a protein required for the spliceosome assembly [43]. Cluster #56 contains the two regulatory subunits of PKA, PRKAR1B, and PRKAR2B, which form a dimer, and several A-kinase scaffold proteins that anchor this dimer to different subcellular compartments [44]. Two altProts interacting with PRKAR2B are also present in this cluster. Interestingly, altProt IP_156019 is encoded by an altORF overlapping the canonical PRKAR2B CDS; hence, *PRKAR2B* is a dual-coding gene with both proteins, the refProt and the altProt, interacting with each other. The discovery of new altProts in known protein communities demonstrates a potential for the increase in our knowledge of biological complexes. We compiled the results of the clustering and GO enrichment into an interactive web application available at https://seb-leb.github.io/altprot-ppi.

### Disease association

**Figure 5:**
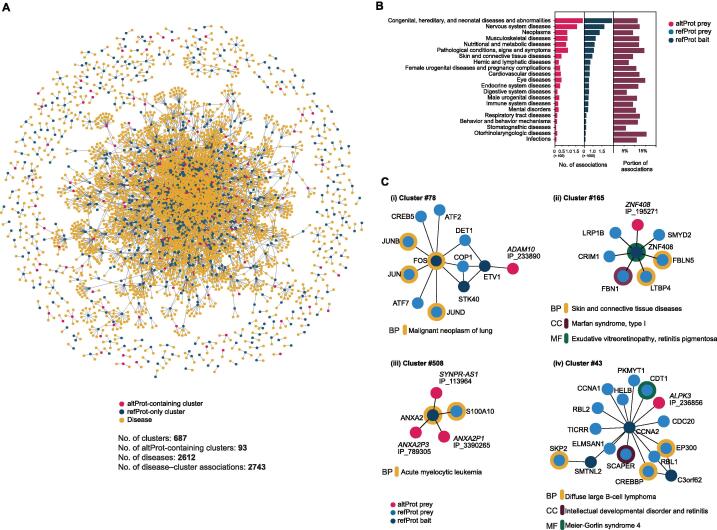
**Communities of proteins with altProt members are associatedwithdisease phenotypes** **A.** Network of associations between protein clusters (blue and red nodes) and diseases (yellow nodes) from DisGenNet. Gene–disease enrichment was computed for each pair of disease–cluster, and associations were deemed significant after hypergeometric test with alpha set to 0.01 and multiple testing correction set at maximum 1% FDR. **B.** Disease–cluster associations counted by disease classification (altProt-containing clusters as red bars, and refProt-only clusters as blue bars) and sorted by portion of associations involving a cluster with altProts (dark red bars). **C.** Focus on clusters with significant disease associations showing involvement of altProts. *ADAM10* is a gene associated with tumorigenesis and produces an altProt here detected as part of a cluster associated to neoplastic processes (i). Other disease–cluster associations include genetic connective tissue diseases involving a pair of proteins encoded by the same gene (ii) and a cluster comprising pseudogene-derived altProts and parental gene refProt in association with another oncological pathology (iii). Cluster #43 highlights associations of a cluster to both rare and common diseases with a community of proteins located at the membrane (iv).

The curated list of disease–gene associations published by DisGeNET relates 6970 genes with 8141 diseases in 32,375 associations [45]. After mapping this disease–gene association network onto our network of protein communities, 687 clusters of which 93 contained at least one altProt were found in association with 2612 diseases (Figure 5A). The 116 disease–cluster associations involving at least one altProt were distributed among 21 disease classes (Figure 5B). The distribution of disease–cluster associations involving altProts among the disease classes was similar to those involving refProts. Thus, no preferential association of altProts with certain disease classes could be observed.

A selection of subnetworks illustrates how altProts associate with different diseases (Figure 5C). *ADAM10* encodes a transmembrane refProt with metalloproteinase activity. Among protein substrates that are cleaved by ADAM10 and shed from cells, some act on receptors and activate signaling pathways important in normal cell physiology [46]. Overexpression of this protease or increased shedding of tumorigenic proteoforms results in overactivation of signaling pathways and tumorigenesis [47], [48]. IP_233890 is an altProt expressed from bicistronic *ADAM10* and its association with a subnetwork of transcription factors involved in tumorigenesis may further clarify the role of that gene in cancer (Figure 5C, i). Cluster #165 illustrates the association of a pair of refProt/altProt expressed from the same dual-coding gene, *ZNF408*, with three different diseases (Figure 5C, ii). The implication of pseudogene-derived altProts is emphasized by the association of three of them with acute myelocytic leukemia through their interaction with *ANXA2* (cluster #508; Figure 5C, iii). Two of these interactions occur between a refProt from the parental gene and altProts encoded by two of its pseudogenes.

Cluster #43 relates proteins that are key regulators of entry into and progression of cell cycle, including at the level of DNA replication and check point control to preserve the integrity of the genome in dividing cells [49], [50]. Through its association with this cluster (Figure 5C, iv), AltProt IP_236856 is likely involved in cell cycle progression and DNA integrity, and characterization of its molecular activity may yield mechanistic insight surrounding associated pathologies.

### Functional validation of PPIs involving an altProt

Interactions representative of the three following classes of complexes involving altProts were selected for further experimental validation: an altProt encoded by a dual-coding gene and interacting with the respective refProt, an altProt expressed from a pseudogene and interacting with the refProt encoded by the parental gene, and an altProt interacting with a refProt coded by a different gene.

**Figure 6:**
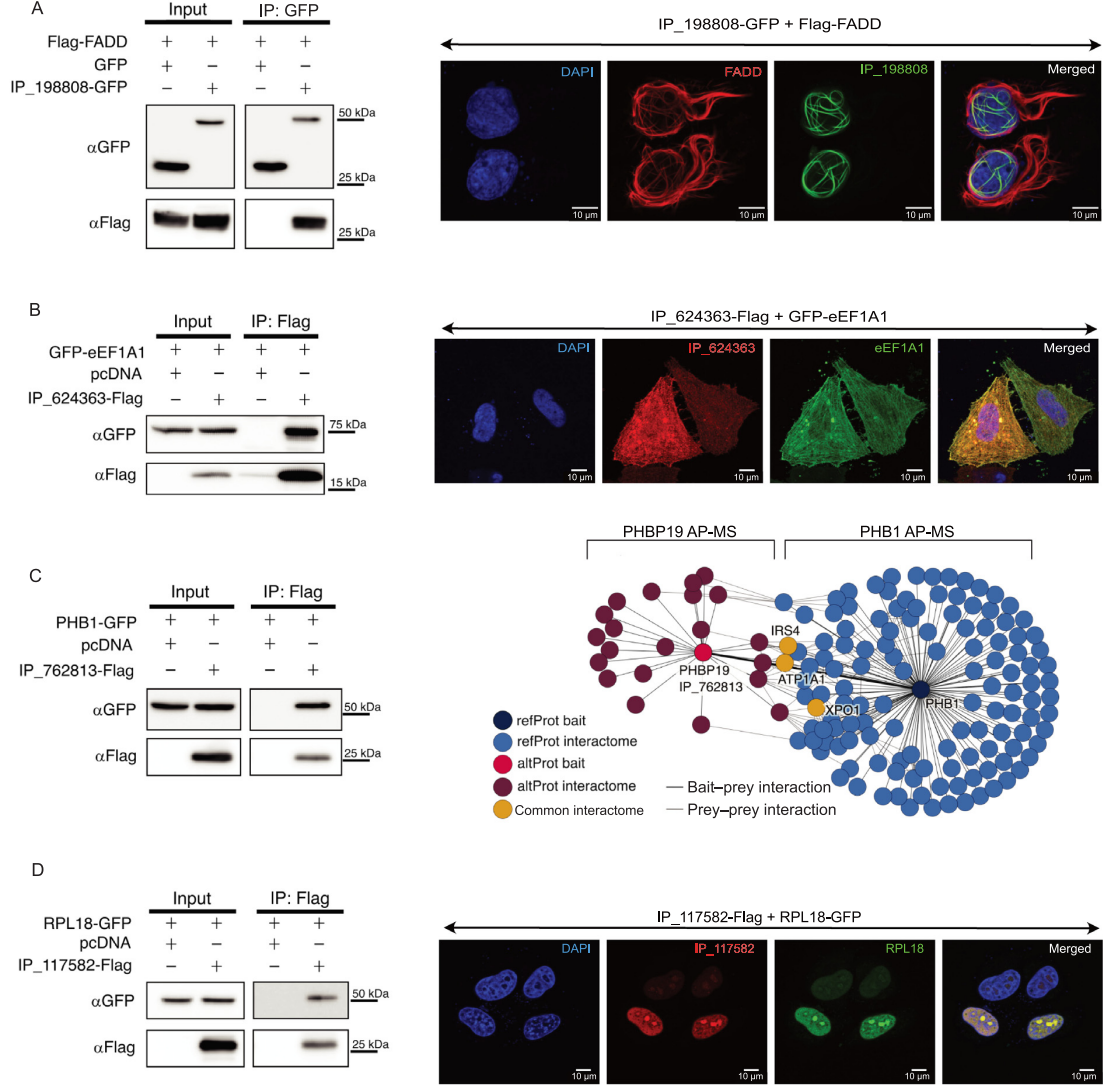
**Experimental validation of refProt–altProt interactions** **A.** Validation of FADD and IP_198808 protein interaction encoded by a bicistronic gene. Left panel: immunoblot of Co-IP with GFP-trap Sepharose beads performed on HEK293 lysates co-expressing Flag-FADD and IP_198808-GFP or GFP only. Right panel: confocal microscopy of HeLa cells co-expressing IP_198808-GFP (green channel) and Flag-FADD (immunostained with anti-Flag; red channel). r = Pearson’s correlation. The associated Manders’ Overlap Coefficients are M1 = 0.639 and M2 = 0.931, respectively. **B.** Validation of eEF1A1 and IP_624363 protein interaction encoded by a pseudogene/parental gene couple. Left panel: immunoblot of Co-IP with Anti-FLAG magnetic beads performed on HEK293 lysates co-expressing GFP-eEF1A1 and IP_624363-Flag or pcDNA3.1 empty vector. Right panel: confocal microscopy of HeLa cells co-expressing GFP-eEF1A1 (green channel) and IP_624363-Flag (immunostained with anti-Flag; red channel). r = Pearson’s correlation. The associated Manders’ Overlap Coefficients are M1 = 0.814 and M2 = 0.954, respectively. **C.** Validation of PHB1 and IP_762813 protein interaction encoded by a pseudogene/parental gene couple. Left panel: immunoblot of Co-IP with Anti-FLAG magnetic beads performed on HEK293 lysates co-expressing PHB1-GFP and IP_762813-Flag or pcDNA3.1 empty vector. Right panel: comparison of the interaction networks of IP_762813-Flag (purple) and PHB1-GFP (blue) from independent AP-MS experiments of both proteins. Three independent AP-MS datasets for each protein. **D.** Validation of RPL18 and IP_117582 protein interaction. Left panel: immunoblot of Co-IP with Anti-FLAG magnetic beads performed on HEK293 lysates co-expressing RPL18-GFP and IP_117582-Flag or pcDNA3.1 empty vector. Right panel: confocal microscopy of HeLa cells co-expressing RPL18-GFP (green channel) and IP_117582-Flag (immunostained with anti-Flag; red channel). r = Pearson’s correlation. The associated Manders’ Overlap Coefficients are M1 = 0.993 and M2 = 0.972, respectively. All Western blots and confocal images are representative of at least three independent experiments. GFP, green fluorescent protein; IP, immunoprecipitation; Co-IP, co-immunoprecipitation.

**Supplementary Figure S4:**
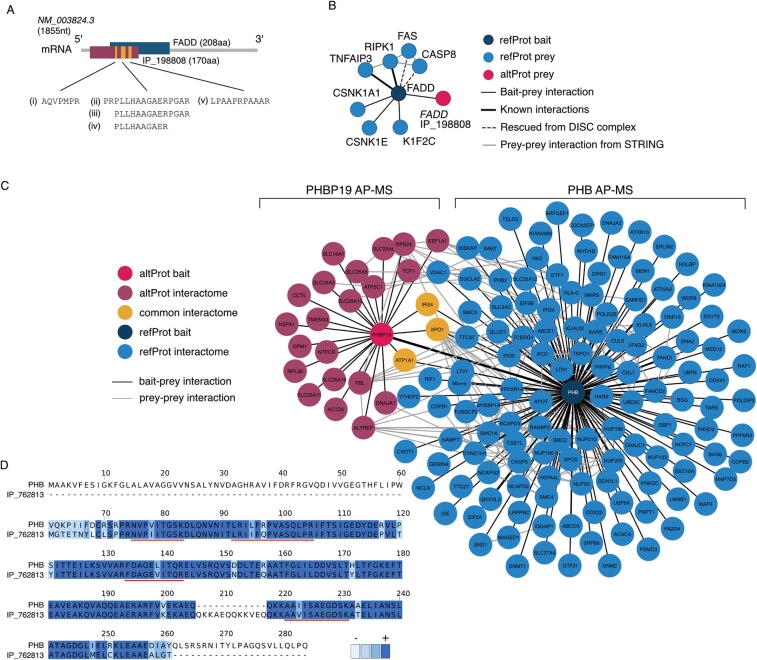
**Validation details A.** Validation of interaction between proteins FADD and IP_198808 encoded by the same mRNA. IP_198808 peptides iii, iv, and v were detected in re-analyses of both ViroTrap and BioPlex 2.0 AP-MS of FADD. Peptides i and ii were exclusively identified in ViroTrap and BioPlex 2.0 re-analyses, respectively. PSMs for i and v from the ViroTrap dataset were validated against unrestricted modifications of refProts using PepQuery. **B.** FADD network after re-analysis of ViroTrap MS data including IP_198808 sequence in the database. **C.** Detailed view of the combined network from AP-MS experiments of PHB refProt and PHBP19 altProt. **D.** Alignment of IP_762813 altProt encoded by pseudogene PHBP19 and PHB1 refProt sequences based on amino acids using Clustalω with default settings. Blue shading indicates amino acid similarity. Unique peptides detected are underlined red.

**Supplementary Figure S5:**
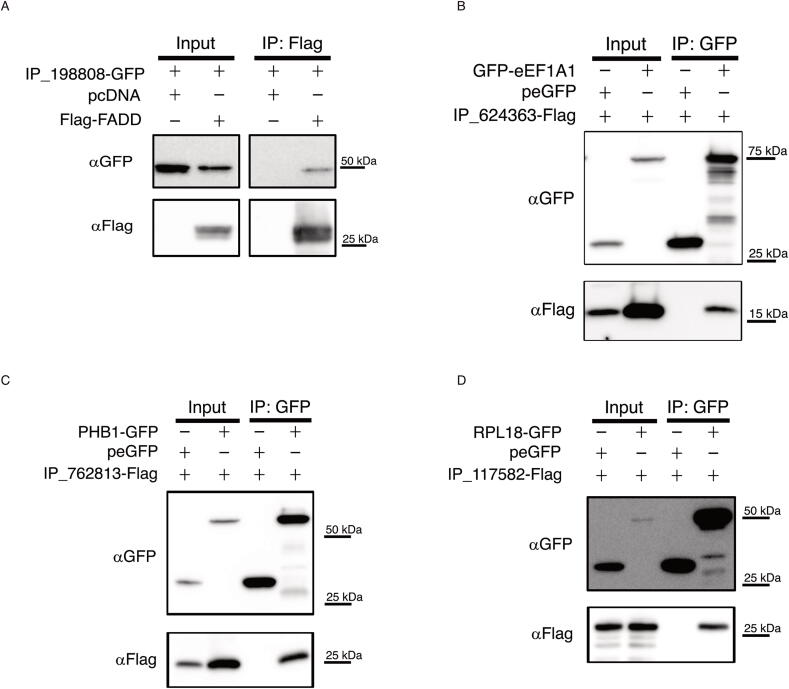
**Complementary co-IP immunoblot validatio**n **A.** Validation of FADD and IP_198808 protein interaction encoded by a bicistronic gene. Immunoblot of co-IP with Anti-FLAG magnetic beads performed on HEK293 nuclear extract co-expressing Flag-FADD and IP_198808-GFP or pcDNA3.1 empty vector with IP_198808-GFP. **B.** Validation of eEF1A1 and IP_624363 protein interaction encoded from a pseudogene/parental gene couple. Immunoblot of co-IP with GFP-trap sepharose beads performed on HEK293 lysates co-expressing GFP-eEF1A1 and IP_624363-Flag or GFP with IP_624363-Flag constructs. **C.** Validation of PHB1 and IP_762813 protein interaction encoded by a pseudogene/parental gene couple. Immunoblot of co-IP with GFP-trap sepharose beads performed on HEK293 lysates co-expressing PHB1-GFP and IP_762813-Flag or GFP with IP_762813-Flag constructs. **D.** Validation of RPL18 and IP_117582 protein interaction. Immunoblot of co-IP with GFP-trap sepharose beads performed on HEK293 lysates co-expressing RPL18-GFP and IP_117582-Flag or GFP with IP_117582-Flag constructs. All western blots and confocal images are representative of at least 3 independent experiments.

The dual-coding *FADD* gene expresses altProt IP_198808 in addition to the conventional FADD protein, and both proteins interact within the DISC complex (Figure 2G, i). We took advantage of a previous study aiming at the identification of the FADD interactome to test whether this altProt may also have been missed in this analysis because the protein database used did not contain altProt sequences [51]. In this work, the authors developed a new method called Virotrap to isolate native protein complexes within extracellular virus-like particles to avoid artifacts of cell lysis in AP-MS. Among the baits under study FADD was selected to isolate the native FADD complex. First, we used the peptide-centric search engine PepQuery to directly test for the presence or the absence of IP_198808-derived specific peptides in the FADD complex datasets. Rather than interpreting all MS/MS spectra, this approach tests specifically for the presence of the queried peptides [52]. Indeed, two unique peptides from IP_198808 were detected in each of the replicates of that study via PepQuery (Figure S4A, peptides i and v). Second, we used a conventional spectrum-centric and database search analysis with the UniProt database to which was added the sequence of IP_198808. The altProt was identified in the FADD interactome (Figure S4B) with 4 unique peptides (Figure S4A, peptides i and iii–v). In cells co-transfected with Flag-FADD and IP_198808-GFP, FADD formed large filaments (Figure 6A, right), previously labeled death effector filaments [53]. IP_198808 co-localized in the same filaments in the nucleus, whereas the cytosolic filaments contained FADD only. Finally, this interaction was validated by reciprocal co-immunoprecipitation (Co-IP) (Figure 6A, left; Figure S5A). These proteomic, microscopic, and biochemical approaches confirmed the interaction between the two proteins encoded in dual-coding *FADD*.

Next, we selected two pairs of interactions of an altProt expressed from a pseudogene with a refProt expressed from the corresponding parental gene. The interaction between altProt IP_624363 encoded in the *EEF1AP24* pseudogene and EEF1A1 (Figure 3A, vi) was confirmed by reciprocal Co-IP from cell lysate from cells co-transfected with GFP-eEF1A1 and IP_624363 (Figure 6B, left; Figure S5B). Both proteins also displayed strong co-localization signals (Figure 6B, right). In order to validate the interaction between *PHBP19*-encoded IP_762813 and PHB1, we performed two experiments. First, PHB1 co-immunoprecipitated with IP_762813 using cell lysates from cells co-transfected with PHB1-GFP and IP_762813-Flag (Figure 6C, left) and the reversed Co-IP was also confirmed (Figure S5C). Second, we performed independent AP-MS experiments for both IP_762813 and PHB1 in HEK293 cells. We confirmed the presence of PHB1 in the interactome of IP_762813 and the presence of IP_762813 in the interactome of PHB1 (Figure 6C, right; Figure S4C and D). Interestingly, we observed shared interactors between IP_762813 and PHB1 [IRS4 (O14654), ATP1A1 (P05023), and XPO1 (O14980)], as well as interactors specific to each. Prey–prey interactions from STRING also showed a certain interconnectivity of both interactomes, whereas each retained unique interactors (Figure 6C, right; Figure S4C). The altProt IP_117582 encoded in the *BEND4* gene is one of the most detected altProt with PSM in seven different pull-downs and three of these interactions were deemed high confidence by the model and integrated our network (Figure 3A, iii). The interaction with RPL18 was tested and confirmed by reciprocal Co-IP in cells co-transfected with RPL18-GFP and IP_117582-Flag (Figure 6D, left; Figure S5D), and their co-localization was also confirmed by immunofluorescence (Figure 6D, right).

## Discussion

The discovery of unannotated altProts encoded by ORFs localized in “non-coding” regions of the transcriptome raises the question of the function of these proteins. The translation of altProts may result from biological translational noise producing non-bioactive molecules. Alternatively, altProts may play important biological roles [11]. Here, we addressed the issue of the functionality of altProts by testing their implication in PPIs. We have re-analyzed the BioPlex 2.0 proteo-interactomics data using the proteogenomics resource OpenProt which provides customized databases for all ORFs larger than 30 codons in 10 species [20], [21]. Under stringent conditions, a total of 278 prey altProts were detected, of which 261 could be confidently mapped in the network of 254 bait refProts. Among them, 117 altProts are expressed from pseudogenes; 118 are expressed from dual-coding and bicistronic genes; and 43 are expressed from transcripts which were annotated as ncRNAs but should in fact be protein-coding. In addition to revealing new members of protein communities, this study lends definitive support to the functionality of hundreds of altProts and provides avenues to investigate their function.

The detection of 278 altProts under stringent conditions confirms the hindrance introduced by three assumptions of conventional annotations: (1) eukaryotic protein-coding genes are monocistronic; (2) RNAs transcribed from genes annotated as pseudogenes are ncRNAs; and (3) ncRNAs are annotated as such based on non-experimental criteria, including the largely used 100 codons minimal length [54]. The persistence of these assumptions in conventional genomic annotations limits the repertoire of proteins encoded by eukaryotic genomes [55]. It remains possible that functional altORFs in regions of the transcriptome annotated as non-coding are exceptions and that a large fraction of genes and RNAs comply with current assumptions. However, an ever-increasing number of proteogenomics studies demonstrate that thousands of altORFs and their corresponding proteins are translated [13], [56].

Conventional annotations introduce some confusion by opting to create a new gene entry within a previously annotated gene where a novel protein product has been reported or where novel transcripts have been mapped, rather than annotate a second ORF in the initial gene. The result is that some genomic regions have been assigned a second gene in the same orientation, nested within a previously annotated gene. This is the case for the pseudogene *ENO1P1* [Ensembl: ENSG00000244457; genomic location: chr1:236,483,165–236,484,468 (GRCh38.p13)] which overlaps with the protein-coding gene *EDARADD* [Ensembl: ENSG00000186197; genomic location: chr1:236,348,257–236,502,915 (GRCh38.p13)] which also encodes altProt IP_079312. Thus, as a result of this annotation, a pseudogene (*ENO1P1*) is nested within a protein-coding gene (*EDARADD*). Similarly, a second protein-coding gene termed *AL022312.1* [Ensembl: ENSG00000285025; genomic location: chr22:39,504,231–39,504,443 (GRCh38.p13)] was added within the protein-coding gene *MIEF1* [Ensembl: ENSG00000100335; genomic location: chr22:39,499,432–39,518,132 (GRCh38.p13)] to annotate the recently discovered altORF upstream of the *MIEF1* CDS [13], [57]. We suggest that recognizing the polycistronic nature of some human genes to be able to annotate multiple protein-coding sequences in the same gene is more straightforward than annotating additional small genes nested in longer genes in order to comply with monocistronic annotations.

The involvement of 261 altProts in 316 of the 19,968 PPIs in the current network represents a sizable number of previously missing nodes and edges and contributes to the understanding of network topology. The impact of altProt inclusion on network structure is revealed by the bridging role many seem to play between interconnected regions (Figure 3A, i–ix). This linkage of otherwise independent complexes introduces major changes to network structure shown to be related to biological system state (*e.g.*, cell type) [9]. Results from the current analysis are thus anticipated to yield insight regarding molecular function and mechanisms of protein complexes in the contexts of cell type and other suborganismally defined states [9]. Indeed, the presence of altProts in protein communities associated with known function and/or diseases makes it possible to generate testable hypotheses regarding their role in physiological and pathological mechanisms [58].

An important observation stemming from the current study is that many pseudogenes encode one altProt in the network, including some encoding two altProts. Strikingly, several altProts expressed from pseudogenes interact with their respective parental protein (more likely to interact compared with any pair of proteins in the network with 45 pairs of pseudogene–parental gene directly interacting out of 107 pairs *vs.* 39,936 direct PPIs of 56,115,693 possible pairs in the networks). This suggests that pseudogene-encoded altProts are functional paralogs and that their incorporation into homomeric protein complexes of the parental protein could modulate or change the activity of the parental complex. Such function would be reminiscent of the role of homomers and heteromers of paralogs in the evolution of protein complexes in yeast, allowing structural and functional diversity [59], [60]. The GAPDH subnetwork with its seven pseudogene-encoded altProts is particularly striking. Besides its canonical function in glycolysis, GAPDH displays a variety of different functions in different subcellular locations, including apoptosis, DNA repair, regulation of RNA stability, transcription, membrane fusion, and cytoskeleton dynamics [38], [39], [61]. We propose that the incorporation of different paralog subunits in this multimeric complex results in the assembly of different heteromeric complexes and may at least in part entail such functional and localization diversity. This hypothesis is in agreement with the speculation that the diversity of functions associated with GAPDH correlates with the remarkable number of GAPDH pseudogenes [37].

Among the genes encoding the 261 altProts inserted in the network, 14 encode refProt/altProt pairs that specifically interact with each other, which implies that these pairs are involved in the same function. Such functional cooperation between a refProt and an altProt expressed from the same eukaryotic gene confirms previous observations in humans [13], [56], [62], [63]. Dual-coding genes are common in viruses [64] and proteins expressed from viral overlapping ORFs often interact [65]. The general tendency of physical or functional interaction between two proteins expressed from the same gene should help decipher the role of newly discovered proteins provided that functional characterization of the known protein is available. Molecular mechanisms behind the functional cooperation of such protein pairs remain to be explored.

Furthermore, several pairs of proteins encoded by the same gene but acting in distant parts of the network have also been identified. Could these altProts be a source of cross talk between functional modules under the same regulation at the genetic level, but multiplexed at the protein function level?

The current study shows that the 261 altProts incorporated in the network differ from refProts by their size (6 times smaller in average), but do not form a particular class of gene products; rather they are members of common communities present throughout the proteomic landscape. Initial serendipitous detection of altProts subsequently called for proteogenomics approaches which widened discoveries via systematic and large-scale detection [66], [67]. System resilience and biodiversity have long been linked in the ecology literature [68]; by analogy the increased proteomic diversity due to altProts could be a contributing factor to this effect in cellular systems. To find out the extent to which altProts play widespread and important biological functions will require more studies in functional genomics.

## Materials and methods

### Classification of proteins, transcripts, and genes

refProts are known proteins annotated in NCBI RefSeq, Ensembl, and/or UniProt. Novel isoforms are unannotated proteins with a significant sequence identity to a refProt from the same gene; for these isoforms, BLAST search yields a bit score over 40 for an overlap over 50% of the queried reference sequence. altProts are unannotated proteins with no significant identity to a refProt from the same gene.

altORFs correspond to unannotated ORFs predicted to encode proteins with no significant identity to any other annotated protein.

We classify RNA transcripts as dual coding or bicistronic based on the relative position of the ORFs on the transcript. If they are overlapping (*i.e.*, if they share nucleotides) we classify the transcript as dual coding, if they are sequential (*i.e.*, if they share no nucleotides) we classify it as bicistronic. Gene classification with this respect is inherited from the classification of transcript that it produces. Note that transcripts and genes can hold both dual coding and bicistronic classifications.

### Re-analysis of AP-MS data

Files obtained from the authors of the BioPlex 2.0 contained the results of 8364 AP-MS experiments using 3033 bait proteins (tagged with GFP) in two technical replicates or more barring missing replicates and corrupted files [4], [5]. Files were converted from RAW to MGF format using ProteoWizard 3.0 and searched with SearchGUI 2.9.0 using an ensemble of search engines (Comet, OMSSA, X!Tandem, and MS-GF+). Search parameters were set to a precursor ion tolerance of 4.5 ppm and fragment ion tolerance of 20 ppm, trypsin digestion with a maximum of two missed cleavages, and variable modifications including oxidation of methionine and acetylation of N termini. The minimum and maximum length for peptides were 8 and 30 aa, respectively. Search results were aggregated using PeptideShaker 1.13.4 with a 0.001% protein-level FDR as described previously [20]. In addition to already annotated proteins, the OpenProt database includes all predicted altProts and novel isoforms. Because large databases result in a large increase of false positive rates [16], [69], this effect is balanced using an FDR of 0.001% at protein level (1% at peptide level) as previously described [18], [19]. The protein library contained a non-redundant list of all refProts (134,477 proteins) from UniProt (release 2019_03_01), Ensembl (GRCh38.95), and RefSeq (GRCh38.p12), in addition to all altProts (488,956 proteins) and novel isoforms (68,612 proteins) predicted from OpenProt 1.6. altProt identifiers throughout the current article are accessions from OpenProt starting with “IP_”. The library was concatenated with reversed sequences for the target decoy approach to spectrum matching.

### Validation of altProt identifications

Novel protein identifications were supported by unique peptides. A minimum of one unique peptide detected in two technical replicas (two injections of the same purifications) was necessary to identify an altProt. A minimum of two unique peptides detected in two technical replica was necessary for the identification of refProts. Because altProts are on average 6 times smaller than refProts (Figure 1D) and thus present less probability of unique peptide detection, a threshold of one unique peptide for altProt identification was deemed necessary. All peptides assigned to altProts are unique matches to the altProt sequence, no non-unique peptides were assigned to altProts. Peptide assignment rules are different for altProts and refProts because more stringent criteria are necessary to confidently identify novel proteins. Unambiguous unique peptides are required for the identification of non-canonical proteins [16].

An additional peptide-centric approach was used to both enforce a significant *P* value on the PSM and validate that spectra supporting such peptides could not be better explained by peptides from refProts with post-translational modifications. PepQuery allows the search of specific peptides in spectra databases using an unrestricted modification search option [22]. All possible peptide modifications from UniMod artifact and post-translational modifications were considered when ensuring unicity of spectral matches (downloaded March 2020) [70].

Because the OpenProt library is derived from the transcriptome as described by annotation of the reference genome, it is possible that genetic variations specific to the cell line used in the BioPlex study (HEK293T) affect the sequences of translated proteins. The sequenced genome of HEK293T [71] was screened to ensure that peptides mapped to altProts did not present single amino acid variations (SAAVs). No variants were found in the regions corresponding to the peptides identifying altProts.

AltProt sequences with peptides validated with PepQuery have been submitted to the UniProt Knowledgebase. All annotated spectra matched to altProt peptides are available in mzIdentML and MGF formats in File S1.

### Synthetic peptide MS/MS analysis

To validate the fragmentation pattern of peptides assigned to altProts in the BioPlex dataset, a set of 100 tryptic peptides from 72 different altProts encoded by transcripts of various biotypes were synthesized (> 50% purity; Biomatik, Ontario, Canada) and subjected to LC-MS/MS analysis.

Two injections were prepared: one containing all synthetic peptides and the other containing a selection of 16 peptides from the first run that were undetected or only resulted in spectra of poor quality. First, the powder was resuspended in a solution of 1% formic acid and 50% acetonitrile. Then, the suspension was diluted to 20 nM in 1% formic acid and 5% acetonitrile prior to injection for the shotgun method (with the same parameters as those in the “MS analysis of in-house affinity purifications” section below) or injection for paired reaction monitoring (PRM) method (as published in [36]). Briefly, peptides were loaded and separated onto a nano high performance liquid chromatography (nanoHPLC) system (Catalog No. Dionex Ultimate 3000, ThermoFisher Scientific, Mississauga, Canada) with a constant flow of 4 μl/min onto a trap column [Acclaim PepMap100 C18 column (0.3 mm id × 5 mm), Dionex Corporation, Sunnyvale, CA]. Peptides were then eluted off toward an analytical column heated to 40 °C [PepMap C18 nano column (75 μm × 25 cm)] with a linear gradient of 5%–45% of solvent B (80% acetonitrile with 0.1% formic acid) over a 42-min gradient at a constant flow (450 nl/min).

Peptides were analyzed on an OrbiTrap Q Exactive (ThermoFisher Scientific) spectrometer using the PRM method. An inclusion list containing the *m/z* values corresponding to the monoisotopic form of the peptides was generated. The collision energy was set at 28% and resolution for the MS/MS was set at 35,000 for 200,000 ions with maximum filling time of 110 ms with an isolation window of 2.0. Data acquisition was conducted with Xcalibur version 4.3.73.11.

PSM was conducted using SearchGUI (version 3.3.17) and PeptideShaker (version 1.16.42) against the Swiss-Prot library (October 1, 2020) of proteins concatenated with the sequences of the 72 altProts (20,431 sequences) with FDR controlled at 1%. PSMs of synthetic peptides were then compared with PSMs observed in the BioPlex dataset using a spectral correlation measure as described by Toprak and his colleagues [72]. An example spectrum comparison (generated with the Universal Spectrum Explorer [73]) as well as an overall summary is available in Figure S2. All synthetic and BioPlex spectral comparisons are provided in Table S2.

### Obtaining spectral counts

Because altProts are smaller than refProts, they have a lower number of uniquely identifying peptides. For this reason, altProts with at least one unique peptide across multiple replicates were considered, but only refProts identified with at least two unique peptides across multiple replicates were retained for downstream analysis. Spectra shared among refProts were counted in the total spectral count of each protein. Spectra assigned to altProts were counted only if unique to the protein or shared with another altProt. Spectra shared between an altProt and at least one refProt were given to the refProt. refProt spectral counts were combined by gene following the methodology of the original study; however, it was necessary to keep altProts separate as many are encoded by genes that already contain a refProt or other altProts.

### Interaction scoring

Following protein identifications, HCIPs were identified following the method outlined in the original study [4]. Briefly, the CompPASS R package was first used to compute statistical metrics (weighted D-score, Z-score, and entropy) of prey identification based on PSM counts. The results from CompPASS were then used to build a vector of nine features (as described by Huttlin and his colleagues [4]) for each candidate bait–prey pair which were passed to a Naive Bayes classifier (CompPASS Plus) tasked with the discrimination of HCIPs from background identifications. The original study also included a class for wrong identification, but because decoy information was unavailable and because our approach employs a FDR three orders of magnitudes lower in the identification step, a third class was not deemed necessary. The classifier was trained in cross-validation fashion using 96 well plate batches as splits and PPIs from the original study as target labels for true interactors.

Threshold selection was implemented considering the Jaccard overlap (*J*), precision, recall, and F1 score metrics [see Equations (1), (2), (3), (4)] between networks resulting from the re-analysis and the original study. The main differences between the OpenProt-derived re-analysis and BioPlex 2.0 lie in the total spectral counts resulting from the use of different search algorithms and more stringent FDR. It was thus important to tune model threshold selection to maximally reproduce results from the original study (Figure S1A). A threshold of 0.045 was selected as it compromised well between optimal Jaccard overlap, F score, and precision (Figure S1B). A summary of protein and interaction counts is shown in Figure S1D.

(1) $$J\left(A,B\right)=\frac{\left|A\cap B\right|}{\left|A\cup B\right|}$$

(2) $$P\mathrm{recision}=\frac{\left|A\cap B\right|}{\left|A\right|}$$

(3) $$R\mathrm{ecall}=\frac{\left|A\cap B\right|}{\left|B\right|}$$

(4) $$F1\;score=2\cdot \frac{P\mathrm{recision}\cdot Recall}{P\mathrm{recision}+Recall}$$

where *A* represents the set of OpenProt-derived PPIs and *B* represents the set of BioPlex 2.0 PPIs.

### Network assembly and structural analysis

Bait–prey pairs classified as HCIPs were combined into an undirected network using genes to represent refProt nodes and OpenProt protein accessions to represent altProt nodes. The NetworkX 2.5 Python package was used for network assembly and all network metrics calculations.

The power law fit to the degree distribution was computed with the discreet maximum likelihood estimator described by Clauset and his colleagues [74].

A list of known protein complexes from CORUM 3.0 [75] (core complexes, downloaded March 2020) was mapped onto the resulting network to assess the validity of identified interactions (Table S5). Only complexes in which at least two subunits corresponded to baits present in the network were selected for downstream analyses. The portion of subunits identified in the direct neighbourhood of baits was computed for each complex.

### Patterns of interactions involving altProts and refProts

We aimed to assess the relationship between pseudogene-derived altProts and their corresponding refProts from parental genes, in terms of their sequence similarity and their degrees of separation in the network. Parental genes of pseudogenes were selected via the psiCube resource [76] combined with manual curation using Ensembl. Needleman–Wunch global alignment algorithm (with BLOSUM62 matrix) as implemented by the sciki-bio Python package (version 0.5.5) was used as a similarity measure between protein sequences.

To assess degrees of separation, shortest path lengths were computed both for altProt–refProt pairs of pseudogene–parental gene and altProt–refProt pairs encoded by the same gene. For the former, when the refProt was not present in the network, or when no path could be computed between nodes, the shortest path length was computed using a mapping of either the BioPlex 2.0 or BioGRID networks [77].

### Community detection via clustering

A Python implementation of the Markov clustering algorithm (https://github.com/GuyAllard/markov_clustering) was used to partition the network into clusters of proteins [41]. Various values of the inflation parameter between 1.5 and 2.5 were attempted and, similarly to the original study, a value of 2.0 was selected as it compared favorably with known protein complexes. Only clusters of three proteins or higher were retained yielding a total of 1054 clusters. Connections between clusters were determined by calculating enrichment of links between proteins in pairs of clusters using a hypergeometric test with alpha value set to < 0.05 and a Benjamini–Hochberg corrected FDR of 1%. A total of 266 pairs of clusters were found to be significantly connected.

### Disease association analysis

A list of 32,375 disease–gene associations curated by DisGeNET (downloaded March 2020) was mapped onto the network of 1054 protein communities. A disease was associated with a cluster when it was deemed enriched in genes associated with the disease as calculated by hypergeometric testing, with alpha value set to < 0.01 and Benjamini–Hochberg corrected FDR of 1%.

### GO enrichment analysis

GO term enrichments for both altProt second neighborhoods and protein clusters were computed using the GOATOOLS Python package (version 1.0.2). Count propagation to parental terms was set to true, with alpha value to 0.05 and a Benjamini–Hochberg corrected FDR of 1%. The set of all nodes in the network was used as background.

### Cloning and antibodies

All nucleotide sequences were generated by the Bio Basic Gene Synthesis service, except for pcDNA3-FLAG-FADD which was gifted by Jaewhan Song (Catalog No. 78802, Addgene plasmid; https://n2t.net/addgene:78802). IP_117582, IP_624363, and IP_762813 were all tagged with 2× FLAG (DYKDDDDKDYKDDDDK) at their C-termini. IP_198808 was tagged with eGFP at its C-terminus. All altProt CDSs were subcloned into a pcDNA3.1 plasmid. The CDSs of RPL18, eEF1A1, and PHB were derived from their canonical transcripts (NM_000979.3, NM_001402.6, and NM_001281496.1, respectively). RPL18 and PHB were tagged with eGFP at their C-termini and eEF1A1 was tagged with eGFP at its N-terminus. All refProt CDSs were subcloned into a pcDNA3.1 plasmid.

### Cell culture, transfection, and immunofluorescence assay

HEK293 and HeLa cultured cells were routinely tested negative for mycoplasma contamination using Universal Mycoplasma Detection Kit (Catalog No. 30–1012K, ATCC, Manassas, VA). Transfection, immunofluorescence, and confocal analyses were carried out as previously described [67]. Transfection was carried out with jetPRIME (Catalog No. CA89129-924, VWR, Toronto, Canada) according to the manufacturer’s protocol unless otherwise stated. For Co-IP assays, a total of 6 µg of DNA per 100-mm dish was transfected consisting of 3 µg of each construct, except for pEGFP, which was transfected under the following conditions: 0.1 µg when co-transfected with IP_117582-Flag and Flag-FADD, 0.3 µg when co-transfected with IP_762813-Flag, and 0.6 µg when co-transfected with IP_624363-Flag to compensate for its higher transfection and expression efficiency. For immunofluorescence assay, cells were fixed in 4% paraformaldehyde for 20 min at 4 °C, solubilized in 1% Triton for 5 min, and incubated in blocking solution (10% Normal Goat Serum in PBS) for 20 min. Primary anti-Flag antibodies (Catalog No. F1804, Millipore Sigma, Etobicoke, Canada) were diluted as 1:1000 in the blocking solution. Secondary anti-mouse Alexa Fluor 647 antibodies (Catalog No. 4410S, Cell Signaling Technology, New England Biolabs, Whitby, Canada) were diluted at 1:1000 in the blocking solution. All images were taken on a Leica TCS SP8 STED 3X confocal microscope.

### Affinity purification and Western blotting

Co-IP experiments via ChromoTek GFP-Trap (Proteintech, Rosemont, IL) were carried out as previously described [13], whereas experiments via Anti-FLAG M2 Magnetic Beads (Catalog No. M8823, Millipore Sigma) were conducted according to the manufacturer’s protocol with minor modifications. Briefly, HEK293 cells were lysed in the lysis buffer (150 mM NaCl, 50 mM Tris pH 7.5, 1% Triton, and 1× EDTA-free Roche protease inhibitors) and incubated on ice for 30 min prior to a double sonication at 12% amplitude for 3 s each (1 min on ice between sonications). The cell lysates were centrifuged, the supernatant was isolated, and the protein content was assessed using BCA assay (Catalog No. PI23223, ThermoFisher Scientific). Anti-FLAG beads were conditioned with the lysis buffer. Then, 20 µl of beads were added to 1 mg of proteins at a final concentration of 1 mg/ml and incubated overnight at 4 °C. Then, the beads were washed four times with the lysis buffer (twice with 800 µl and twice with 500 µl) prior to elution in 45 µl of Laemmli buffer and boiled at 95 °C for 5 min. For Co-IP of PHB1-GFP and RPL18-GFP, stringent wash was done with modified lysis buffer [250 mM NaCl with 20 µg/ml peptide FLAG (Catalog No. F3290, Millipore Sigma)] prior to elution with 200 µg/ml peptide FLAG. Eluates were loaded onto 12% SDS-PAGE gels for Western blotting of GFP- and FLAG-tagged proteins. 40 µg of input lysates were loaded into gels as inputs. Western blotting was carried out as previously described [67]. The primary antibodies were diluted as follows: anti-Flag (1:1000; Catalog No. F7425, Millipore Sigma) and anti-GFP (1:8000; Catalog No. sc-9996, Santa Cruz, Dallas, TX). The secondary antibodies were diluted as follows: anti-mouse HRP (1:10000; Catalog No. sc-516102, Santa Cruz) and anti-rabbit HRP (1:10000; Catalog No. 7074S, Cell Signaling Technology).

### Affinity purification of nuclear extracts

For Co-IP with GFP beads of Flag-FADD and IP_198808-GFP, nuclear extracts were used instead of cells lysate because the interaction was exclusively observed in the nucleus by confocal microscopy. Nuclear extracts were prepared as previously described [78]. Briefly, HEK293a cells were lysed in Buffer A (10 mM HEPES pH 7.9, 10 mM KCl, 1.5 mM MgCl_2_, 0.34 M sucrose, 10% glycerol, 1 mM DTT, 0.1% Triton, and 1× EDTA-free Roche protease inhibitors) and incubated on ice for 8 min before centrifugation at 1300 *g* for 5 min to remove cytoplasmic soluble proteins (supernatant). The pellet was resuspended with Buffer B (3 mM EDTA, 0.2 mM EGTA, and 1× EDTA-free Roche protease inhibitors) and incubated for 30 min on ice prior to centrifuge at 1700 *g* for 5 min. The supernatant containing nuclear proteins was used for Co-IP. 1/50 volume was kept for input and the remaining was used with anti-FLAG conditioned beads and incubated for 2 h at 4 °C with agitation. The volume was adjusted to 1 ml with lysis buffer (see the “Affinity purification and Western blotting” section). Then, the beads were washed four times with lysis buffer (twice with 800 µl and twice with 500 µl) prior to elution with 30 µl of glycine (0.1 M pH 3.0), 10 min agitation, and stopped with 6 µl Tris (1 M pH 8.0). Eluates were loaded onto 12% SDS-PAGE gels for Western blotting (see the “Affinity purification and Western blotting” section for details).

### AP-MS

For interactome analysis by MS, HEK293 cells at a 70% confluence were transfected with GFP-tagged PHB or with FLAG-tagged PHBP19 (IP_762813). After 24 h of transfection, cells were rinsed twice with PBS, and lysed in the AP lysis buffer (150 mM NaCl, 50 mM Tris-HCl, and 1% Triton). Protein concentration was evaluated with a BCA dosage and 1 mg of total protein was incubated at 4 °C for 4 h with agarose ChromoTek GFP beads (Proteintech) for PHB-GFP or with magnetic FLAG beads (Catalog No. M8823, Millipore Sigma) for IP_762813-Flag. The beads were pre-conditioned with the AP lysis buffer. The beads were then washed twice with 1 ml of AP lysis buffer, and 5 times with 5 ml of 20 mM NH_4_HCO_3_ (ABC) (Catalog No. A6141, Millipore Sigma). Proteins were eluted and reduced from the beads using 10 mM DTT with 15 min at 55 °C, and then treated with 20 mM IAA for 1 h at room temperature in the dark. Proteins were digested overnight by adding 1 μg of trypsin (Promega, Madison, WI) in 100 μl ABC at 37 °C overnight. Digestion was quenched using 1% formic acid and the supernatant was collected. Beads were washed once with acetonitrile/water/formic acid (1/1/0.01 v/v) and pooled with supernatant. Peptides were dried with a speedvac, desalted using a C18 Zip-Tip (Millipore Sigma), and resuspended into 30 μl of 1% formic acid in water prior to MS analysis.

### MS analysis of in-house affinity purifications

Peptides were separated in a PepMap C18 Nano Column (75 μm × 50 cm; ThermoFisher Scientific). The setup used a 0%–35% gradient (0–215 min) of 90% acetonitrile, 0.1% formic acid at a flow rate of 200 nl/min followed by acetonitrile wash and column re-equilibration for a total gradient duration of 4 h with a Ultimate 3000 RSLC (ThermoFisher Scientific). Peptides were sprayed using an EASY-Spray Source (ThermoFisher Scientific) at 2 kV coupled to a quadrupole-Orbitrap (Q Exactive, ThermoFisher Scientific) mass spectrometer. Full-MS spectra within a *m/z* 350–1600 mass range at 70,000 resolution were acquired with an automatic gain control (AGC) target of 1E6 and a maximum accumulation time (maximum IT) of 20 ms. Fragmentation (MS/MS) of the top ten ions detected in the Full-MS scan at 17,500 resolution, AGC target of 5E5, and a maximum IT of 60 ms with a fixed first mass of 50 within a 3 *m/z* isolation window at a normalized collision energy (NCE) of 25. Dynamic exclusion was set to 40 s. MS RAW files were searched with the Andromeda search engine implemented in MaxQuant 1.6.9.0. The digestion mode was set at Trypsin/P with a maximum of two missed cleavages per peptides. Oxidation of methionine and acetylation of N-terminal were set as variable modifications, and carbamidomethylation of cysteine was set as fixed modification. Precursor and fragment tolerances were set at 4.5 and 20 ppm, respectively. Files were searched using a target-decoy approach against UniProt Knowledgebase (*Homo sapiens*, Swiss-Prot, released in October 2020) with the addition of IP_762813 sequence for a total of 20,360 entries. The FDR was set at 1% for PSM, peptide, and protein levels. Only proteins identified with at least two unique peptides were kept for downstream analyses.

### HCIP scoring of in-house affinity purifications

Protein interactions were scored using the SAINT algorithm [79]. For each AP-MS, experimental controls were used: GFP alone-transfected cells for PHB-GFP AP and mock-transfected cells for IP_762813-2F AP. For the PHB-GFP AP, controls from the CRAPome repository [80] corresponding to transient GFP-tag expression in HEK293 cells, pulled using camel agarose beads were used. These controls are: CC42, CC44, CC45, CC46, CC47, and CC48. For the IP_762813-Flag AP, controls from the CRAPome repository [80] corresponding to transient FLAG-tag expression in HEK293 cells, pulled using M2-magnetic beads were used. These controls are: CC55, CC56, CC57, CC58, CC59, CC60, and CC61. The fold-change over the experimental controls (FC_A), over the CRAPome controls (FC_B), and the SAINT probability scores were calculated as follows. The FC_A was evaluated using the geometric mean of replicates and a stringent background estimation. The FC_B was evaluated using the geometric mean of replicates and a stringent background estimation. The SAINT score was calculated by SAINTexpress using experimental controls and default parameters. Proteins with a SAINT score above 0.8, a FC_A and a FC_B above 1.5 were considered HCIPs.

### Network visualization of in-house affinity purifications

The network was built using Python scripts (version 3.7.3) and the NetworkX package (version 2.4). The interactions from the STRING database were retrieved from their protein links downloadable file. Only interactions with a combined score above 750 were kept.

## Code availability

The Python scripts and notebooks containing the analyses are available at https://github.com/Seb-Leb/altProts_in_communities.

## Data availability

The protein interaction AP-MS data for both IP_762813 and PHB1 in HEK293 cells are deposited to the ProteomeXchange Consortium via the PRIDE [81] partner repository (ProteomeXchange: PXD02249), and are publicly accessible at http://proteomecentral.proteomexchange.org/cgi/GetDataset?ID=PXD02249.

## Competing interests

The authors have declared no competing interests.

## CRediT authorship contribution statement

**Sébastien Leblanc:** Conceptualization, Investigation, Visualization, Data curation, Formal analysis, Writing – original draft, Writing – review & editing. **Marie A. Brunet:** Conceptualization, Investigation, Writing – review & editing. **Jean-François Jacques:** Investigation, Writing – review & editing. **Amina M. Lekehal:** Investigation. **Andréa Duclos:** Investigation. **Alexia Tremblay:** Investigation. **Alexis Bruggeman-Gascon:** Investigation. **Sondos Samandi:** Project administration, Supervision, Writing – review & editing. **Mylène Brunelle:** Project administration, Supervision. **Alan A. Cohen:** Formal analysis, Writing – review & editing. **Michelle S. Scott:** Formal analysis, Writing – review & editing. **Xavier Roucou:** Conceptualization, Resources, Funding acquisition, Project administration, Supervision, Writing – original draft, Writing – review & editing. All authors have read and approved the final manuscript.

## Supplementary material

Supplementary File S1 **BioPlex altProt spectra**
